# Efficient cluster-based routing protocol for wireless sensor networks by using collaborative-inspired Harris Hawk optimization and fuzzy logic

**DOI:** 10.1371/journal.pone.0301470

**Published:** 2024-04-05

**Authors:** Huangshui Hu, Xinji Fan, Chuhang Wang

**Affiliations:** 1 College of Computer Science and Engineering, Changchun University of Technology, Changchun, China; 2 College of Computer Science and Technology, Changchun Normal University, Changchun, China; University 20 Aout 1955 skikda, Algeria, ALGERIA

## Abstract

In wireless sensor networks, the implementation of clustering and routing protocols has been crucial in prolonging the network’s operational duration by conserving energy. However, the challenge persists in efficiently optimizing energy usage to maximize the network’s longevity. This paper presents CHHFO, a new protocol that combines a fuzzy logic system with the collaborative Harris Hawks optimization algorithm to enhance the lifetime of networks. The fuzzy logic system utilizes descriptors like remaining energy, distance from the base station, and the number of neighboring nodes to designate each cluster head and establish optimal clusters, thereby alleviating potential hot spots. Moreover, the Collaborative Harris Hawks Optimization algorithm employs an inventive coding mechanism to choose the optimal relay cluster head for data transmission. According to the results, the network throughput, HHOCFR is 8.76%, 11.73%, 8.64% higher than HHO-UCRA, IHHO-F, and EFCR. In addition, he energy consumption of HHOCFR is lower than HHO-UCRA, IHHO-F, and EFCR by 0.88%, 39.79%, 34.25%, respectively.

## 1. Introduction

Wireless Sensor Networks (WSNs) are widely utilized in environment monitoring [1], path planning [2], real-time monitoring [3], healthcare [4], industrial automation [5], and diverse domains owing to their capacity to operate effectively in harsh and remote environments. Comprised of numerous small, cost-effective sensor nodes, these networks have restricted processing, storage, and energy capacities. Due to their deployment in remote areas, replacing power sources or recharging nodes is often impractical. Effectively organizing and managing all nodes is vital to extend the network’s lifetime and enhance task performance. In recent decades, several clustering and routing protocols have arisen as highly effective solutions to tackle this challenge [6–8].

The cluster routing protocol for wireless sensor networks aims to organize nodes into clusters, designating specific nodes as cluster heads (CHs) responsible for managing and transmitting data within each cluster. These CHs play a crucial role in aggregating and relaying data to the base station (BS), thereby reducing network traffic and energy consumption. The well-known Low Energy Adaptive Clustering Hierarchy (LEACH) [9], initially proposed by Heinzelman et al. and later enhanced in [10], heavily relies on random selection and probability calculations for CHs selection. However, this approach can rapidly deplete the energy of CHs, cause uneven energy distribution among network nodes, and overlook adequate load balancing, potentially leading to network overload in specific areas and affecting overall performance. In response, scholars have proposed various enhanced CH selection and routing path determination algorithms, often employing intelligent computing methods such as fuzzy logic [11], genetic algorithms [12], particle swarm optimization [13], ant colony optimization [14], and combinations like fuzzy logic with particle swarm optimization [15], fuzzy logic with ant colony optimization [16], and fuzzy logic with weed optimization [17]. Particularly, fuzzy logic stands out for its ability to handle uncertainties inherent in clustering and routing compared to other algorithms [18,19]. Moreover, it offers greater flexibility than explicit logic, enabling optimal solutions by combining input parameters more effectively.

Despite some progress, optimizing cluster routing in wireless sensor networks remains a complex NP-hard problem, necessitating further exploration to minimize energy consumption and achieve network load balance. The Harris Hawk Optimization (HHO) [20], inspired by hawks’ foraging behavior, displays promising potential in addressing optimization challenges and has found application in clustering [21], routing [22], path planning [23], and WSN clustering-routing dilemmas [24]. Moreover, the advantages of the improved algorithm for Harris Hawk over the original HHO algorithm have been demonstrated in various fields [25,26].

### 1.1. Contributions

This paper proposes the Collaborative Harris Hawk Fuzzy Optimization (CHHFO) method, rooted in a fuzzy logic system (FLS) and collaborative behavior, to enhance the network’s clustering efficiency and CH node selection mechanism. Leveraging fuzzy logic and collaboration, the CHHFO protocol promotes algorithmic performance, refining clustering solutions. Extensive simulations illustrate that the CHHFO-based clustering method outperforms various advanced clustering-routing techniques in the network lifecycle, throughput, and energy consumption. The primary contributions are outlined below.

➢ Utilizing the Mamdani fuzzy inference system, the selection of the optimal CH node relies on factors such as residual energy, distance to the base station, and the count of neighboring nodes.
➢ The determination of the optimal relay node is achieved through the collaborative Harris Hawk optimization algorithm, introducing a novel adaptive function to assess the Harris Hawk’s efficacy while considering energy consumption and load balance.
➢ The performance evaluation of the proposed CHHFO protocol is conducted through comprehensive simulations, demonstrating its superiority over existing methods from various metrics.

Section 2 discusses related works focusing on clustering and routing protocols that leverage fuzzy logic and intelligent algorithms. Section 3 outlines the system model and associated terminology. Section 4 provides a detailed description of the proposed CHHFO protocol. Following that, Section 5 assesses the performance of CHHFO. Finally, Section 6 concludes the paper and indicates potential future research directions.

## 2. Related works

LEACH is a typical wireless sensor network protocol, which works on the principle of prolonging the network lifetime through cluster formation and hierarchical management. LEACH uses clustering to organize the sensor nodes into multiple clusters, and each cluster has a cluster head responsible for management and communication. In order to reduce the energy consumption, LEACH adopts the strategy of rotating cluster heads so that each node becomes a cluster head with a certain probability in order to share the energy burden. However, this random and probability-based cluster head selection may lead to rapid energy depletion of some nodes, triggering an uneven energy distribution in the network. These limitations of the LEACH protocol provide a critical background for the subsequent proposal of new approaches, which motivates further in-depth research in academia to seek better energy management and load balancing solutions for wireless sensor networks. These efforts include the introduction of fuzzy logic and intelligent algorithms to improve node selection strategies, optimize the management of cluster heads, and data transmission to address energy exhaustion and load imbalance.

In LEACH-ERE [27], a probabilistic based mechanism like LEACH is used to select the candidate CHs at first, then the candidates use fuzzy logic to calculate the chance of being a CH considering two inputs residual energy and expected residual energy. The LEACH-ERE protocol can effectively improve the network lifetime. However, it may result in non-uniformly distributed clusters due to its probabilistic selection of the candidate CHs and neglect of other parameters. In contrast, Mamdani fuzzy system is introduced in LEACH-FL [28] to select CH directly. In each round, the fuzzy logic system in each node takes battery level of node, node density and distance from BS into consideration, which means more higher battery level of node, denser the node density, and closer distance from BS, the greater the probability the node becomes CH. After fuzzy inference based on IF-THEN rules and the inputs, the final crisp output probability value can be obtained by defuzzification of the center of area (COA). Different from LEACH selecting CH in a probabilistic manner by comparing a randomly given number with a threshold value, LEACH-FL compares the probability value with the threshold value defined in LEACH, and if it is less than the threshold value, the node becomes a CH, otherwise, it becomes a CM. The next steps are the same as LEACH, clusters are formed by message interaction between CMs and CH, and data transmission is achieved by using TDMA. Simulation results indicate that LEACH-FL outperforms LEACH in terms of network energy consumption and lifetime. However, the LEACH-FL method of selecting cluster heads tends to lead to energy voids close to the base station thus affecting the network lifetime, and in order to solve this problem, the Energy-Aware Unequal Clustering with Fuzzy algorithm (EAUCF) was proposed in [29]. EAUCF is a distributed competition algorithm, which first uses a probability model to select a tentative cluster head, and then selects the final cluster head based on energy competition. In order to solve the hot spots problem, EAUCF allocated an appropriate range of competition for the tentative cluster heads and carried out unequal clustering. EAUCF takes the remaining energy of the node and the distance to the base station as fuzzy inputs, and calculates the competition radius through fuzzy logic. The radius adjustment mechanism of the algorithm makes the cluster close to the base station smaller, reduces the workload in the cluster, solves hot issues well, and prolongs the life of the network. However, EAUCF did not consider the density of nodes in the cluster head election stage, and did not solve the problems of hot spots and energy holes in dynamic networks. In order to solve the problem of node density which is not considered in EAUCF algorithm, EFCR [30] introduces the number of neighboring nodes of a node in the fuzzy input parameter part, at the same time, EFCR algorithm also adds the node’s distance to the base station and the node’s residual energy as the two parameters affecting the node’s energy consumption, and selects the node with the highest degree of cluster-head adaptation for each area as the cluster head in data transmission phase, and collects the data transmitted by non-cluster-head nodes. The transmitted data is fused and sent to the base station. Secondly, in order to reduce the unnecessary node energy consumption caused in each round of clustering, the EFCR algorithm uses the optimal cluster heads selected in the previous round for data transmission and uses a threshold value to determine whether the next round needs to be re-clustered. Simulation results show that EFCR outperforms other algorithms in terms of residual energy, number of surviving nodes and network lifetime. However, the cluster routing approach of fuzzy logic systems alone sometimes fails to adapt to changing network.

With the in-depth research on intelligent optimization algorithms, many scholars have applied various algorithms such as PSO, GWO and HHO to help optimize the performance and energy consumption of wireless sensor networks. Similar to EFCR, [31] introduces a PSO-based multi-hop clustering routing algorithm called EBPSO, which establishes an appropriate energy threshold in order to minimize the energy consumption and to distinguish it from most of the clustering routing protocols that use rounds-based policy (RBP). Re-clustering is performed only when the energy of the clustered nodes is lower than this threshold to prevent excessive energy consumption due to frequent clustering. In the initial stage of CH selection, the most appropriate CH is chosen by considering the energy of the sensor nodes, the distance between the nodes in the cluster, and the distance between the CH and the BS.In the subsequent stage of data transmission, a relay node selection mechanism is introduced to prioritize the nodes with higher residual energies and closer to the BS as the relay nodes. Simulation results show that the wireless sensor network protocol incorporating intelligent algorithms also performs relatively well. Similarly, [32] proposed an energy efficient cluster head selection algorithm EECHIGWO based on the GWO algorithm to improve energy efficiency, average throughput, network stability and network lifetime of wireless sensor networks. The algorithm is defined in terms of rounds and each round consists of CH formation phase and data transmission phase. The fitness value of a node is calculated based on the residual energy and its distance to the BS.The algorithm determines and uniformly selects the CHs in each round which leads to balanced energy consumption and avoids premature death of nodes. Similarly, HHO is heavily used due to its superior performance. In HHO-UCRA [33], a HHO algorithm is introduced to solve the hot spot problem. The algorithm first proposes a novel fitness function which takes into account factors such as neighbor node distance, distance to BS and energy ratio. The HHO algorithm uses this function to select the best sensor node as CH. Subsequently, the CH selection process takes into account factors such as residual energy of the cluster head, nodality of the cluster head, distance between the cluster head and the sensor node as well as the distance between the BS and the CH. Then unequal clusters are formed and sensors are assigned to CHs. Finally, after creating these unequal clusters, an optimized energy efficient multihop routing approach is used for data forwarding using HHO algorithm considering factors such as residual energy of the successor CH, distance from the successor node to the BS and node degree of the successor CH node. As research has progressed, more and more researchers have centred their studies on combining the two approaches.

In addition to traditional intelligent algorithms, approaches that include fuzzy logic have shown good results in solving energy consumption and load balancing problems in wireless sensor networks. Fuzzy logic has advantages in dealing with uncertainty, while intelligent algorithms can optimize parameter selection and node management. In recent years, researchers have begun to explore how to combine fuzzy logic with particle swarm optimization, Harris Hawk algorithm and other intelligent algorithms. This fusion can better handle the complexity and uncertainty in networks and provide better solutions for node selection and data transmission. In [34], an on-demand fuzzy clustering algorithm is presented to improve the network energy efficiency and throughput. At first, a new threshold function is defined to probably select candidate CHs considering more parameters than LEACH including nodes’residual energy and optimal number of clusters, which ensures that the responsibility of being a CH gets rotated among all the nodes and the nodes with higher residual energy than other nodes are elected to be CHs. In order to improve the network performance, a Mamdani fuzzy logic system is used to calculate chance for each node, which uses node degree, node centrality and packet drop probability as descriptors. Moreover, PSO with fitness function maximizing the chance value is adopted to obtain the best ranges of membership functions for inputs and output of the fuzzy logic system. In the end, a candidate CH with higher chance value is selected as the final CH. Once the final CHs are selected, the other nodes become cluster member and join the nearest CH based on received signal strength. For data transmission, the CMs transmit sensed data to their respective CH based on TDMA scheme, and the CHs receive and aggregate the data and forward it to the BS. Finally, only when CHs’residual energy is lower than a threshold defined by *γE*_*initial*_(0<*γ*<1) (*E*_*initial*_ is the initial energy of nodes), the CH sends a message to the BS who is responsible for informing all the nodes to perform re-clustering. Simulation results show that the proposed algorithm can reduce the network energy consumption and improve packet delivery ratio. [35] proposes an energy efficient clustering and routing method based on fuzzy logic and PSO (FLPSOC), which employs a fuzzy logic system for initial CH selection. The input parameters include the ratio of initial energy to residual energy, the number of neighbors and the distance between neighbors. Subsequently, a PSO algorithm based on a fitting function of the residual energy of CHs and the distance between CHs and BSs is used to find the optimal relay CHs. simulations validate the effectiveness of the algorithm in terms of network lifetime, throughput, stability period, and number of cluster heads. To improve the network lifetime. [36] proposed a fuzzy based Improved Harris Hawk Optimization Algorithm (IHHO-F). The algorithm aims to determine the optimal set of CHs through two phases: exploration and exploitation. The strategy uses differential evolution to converge the solution set and generate P*. In each round, the node closest to P* represents the solution set containing CHs. Subsequently, fuzzy logic is employed to select the optimal CHs. Similarly, the solutions are grouped into clusters using a fuzzy logic system to determine the optimal set of cluster heads based on three parameters: the current energy of the CH node, the distance from the cluster head to the convergence point, and the number of neighboring nodes of the CH. A node is designated as Super Cluster Head (SCH) if it has higher residual energy, is closer to the BS and has a higher density of neighboring nodes. In the data transmission phase, the CH collects data from the member nodes. If the hop count is 1, it transmits the data directly to SCH. Otherwise, it routes the data through intermediate CHs. Subsequently, the SCH collects data from the respective CHs using a time-sharing method and forwards it to the BS. Simulation results validate the effectiveness. Comparison of different protocols related to the proposed CHHFO is shown in Table 1.

**Table 1:** Comparison of protocols.

*Protocol*	*Subject*				*Objectives*
	*Clustering*		*Routing*		
	*Method*	*Parameters*	*Method*	*Parameters*	
*LEACH-ERE*	*Mamdani fuzzy system*	*Residual energy and expected residual energy*	*-*	*-*	*Collect information more efficiently*
*LEACH-FL*	*Mamdani fuzzy system*	*Battery level of node, node density and distance from BS*	*-*	*-*	*Network energy* *consumption and lifetime*
*EAUCF*	*Mamdani fuzzy system*	*The remaining energy of the node and the distance to the base station*	*-*	*-*	*Prolongs the life of the network*
*EFCR*	*Mamdani fuzzy system*	*Node’s distance to the base station, the node’s residual energy and the number of neighboring nodes*	*Mamdani fuzzy system*	*Node’s distance to the base station, the node’s residual energy and the number of neighboring nodes*	*Prolongs the life of the network*
*EBPSO*	*PSO*	*The energy of the sensor nodes, the distance between the nodes in the cluster, and the distance between the CH and the BS*	*Relay node selection mechanism*	*Residual energies and distance to the BS*	*Minimize the energy consumption*
*EECHIGWO*	*GWO*	*Residual energy and distance to the BS*	*GWO*	*Residual energy and distance to the BS*	*Improve energy efficiency*
*HHO-UCRA*	*HHO*	*Residual energy of the cluster head, nodality of the cluster head, distance between the cluster head and the sensor node as well as the distance between the BS and the CH*	*HHO*	*Residual energy* *of the successor CH, distance from the successor node to the BS and node degree of the successor CH node*	*Solve the hot spot problem*
*[34]*	*Mamdani fuzzy system*	*Node degree, node centrality and packet drop probability*	*PSO*	*Chance*	*Improve the network energy efficiency and throughput*
*FIPSOC*	*Mamdani fuzzy system*	*The ratio of initial energy to residual energy, the number of neighbors and the distance between neighbors*	*PSO*	*Residual energy of CHs and the distance between CHs and BS*	*Prolongs the life of the network*
*IHHO-F*	*I-HHO*	Uses differential evolution to converge the solution set and generate P*	*Mamdani fuzzy system*	*The current energy of the CH node, the distance from the cluster head to the convergence point, and the number of neighboring nodes of the CH*	*Improve the network lifetime*
*Our proposed*	*Mamdani fuzzy system*	*Residual energy, number of neighboring nodes and distance to BS*	*CHHO*	*Fitness function*	*Maximize the network’s longevity*

## 3. System model

### 3.1. Network model

CHHO, akin to references [9,33,37], utilizes the prevalent network model. This model portrays a substantial number of nodes randomly spread across the detection area, with a single static BS positioned at the area’s center (The BS’s placement can be anywhere within the detection area). The BS manages data collection from the CHs, which, in turn, receive data from the CMs responsible for sensing. The CM’s duty involves collecting the detection data. Furthermore, we establish certain assumptions concerning the network.

- BS’s energy is not limited;
- All nodes have their own *IDs* for identification purposes;
- Each node has the same initial state;
- Nodes are freely distributed and can adjust their firing frequency.

### 3.2. Energy model

**Fig 1:**
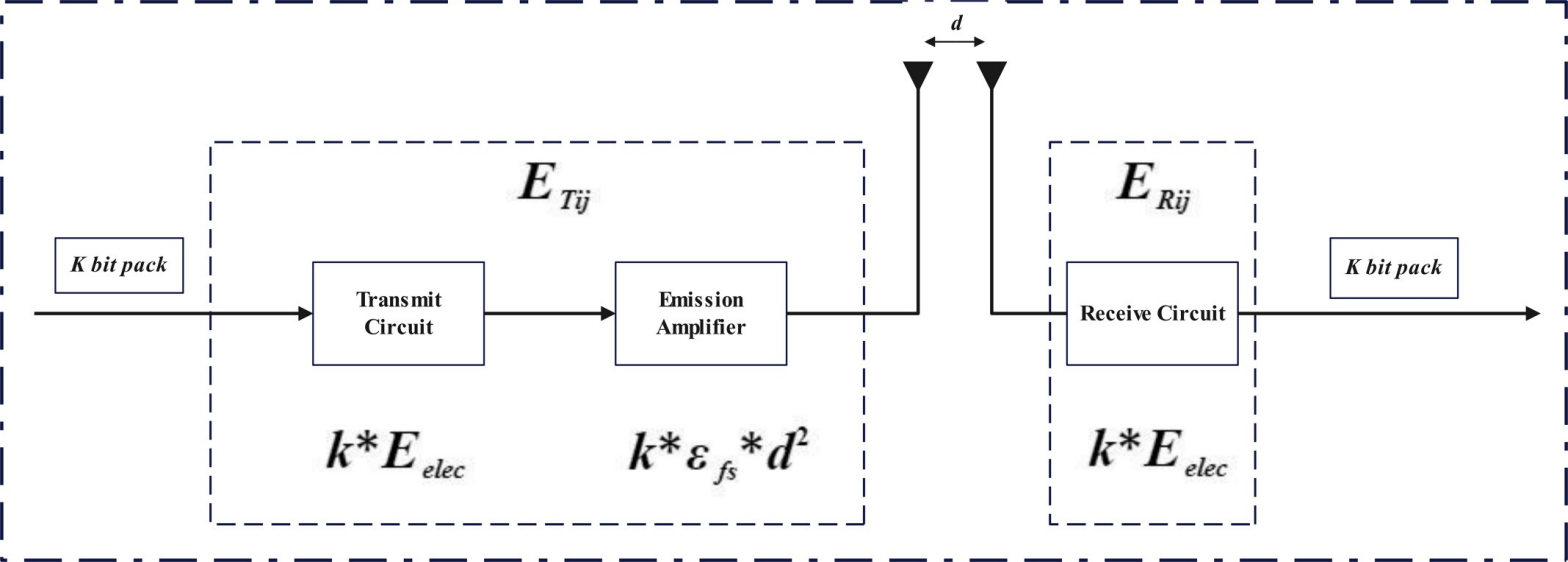
Radio energy consumption model.

To analyze the network energy consumption, the first-order radio model is used to calculate the energy consumption of the transmitter and receive, as shown in Fig 1. The energy model in CHHFO is the same as in [9,35] which is separated into two channels the free space and multi-path fading based on the distance between the transmitter and receiver. When the distance is greater than a threshold value *d*_0_, the multi-path model is adopted, otherwise the free space model is utilized. Thus if a node *i* sends *k*-bit data to a node *j* far away from the distance *d*, the energy required is given as [31]:

$$E_{Tij}=\left\{ \begin{array}{c} k*E_{elec}+k*\varepsilon_{fs}*d^{2},d<d_{0}\\ k*E_{elec}+k*\varepsilon_{mp}*d^{4},d\geq d_{0} \end{array}\right.$$ (1)

Where *E*_*elec*_ represents the electronics energy consumption used for transmitting or receiving 1-bit data. *ε*_*fs*_ and *ε*_*mp*_ are the amplifier coefficients used for free space and multi-path model, and $d_{0}=\sqrt{\varepsilon_{fs}/\varepsilon_{mp}}$ is the distance threshold for the model determination. Besides, the energy consumed for *k*-bit data aggregation can be calculated by using Eq (2) [31,37].


$$E_{Rij}=k*E_{elec}$$ (2)

Besides, the energy required for aggregating *k*-bit data is given by:

$$E_{DA}=k*E_{pDb}$$ (3)

Where *E*_*pDb*_ denotes the energy consumption for 1-bit data fusion [32].

## 4. Propose protocol

**Fig 2:**
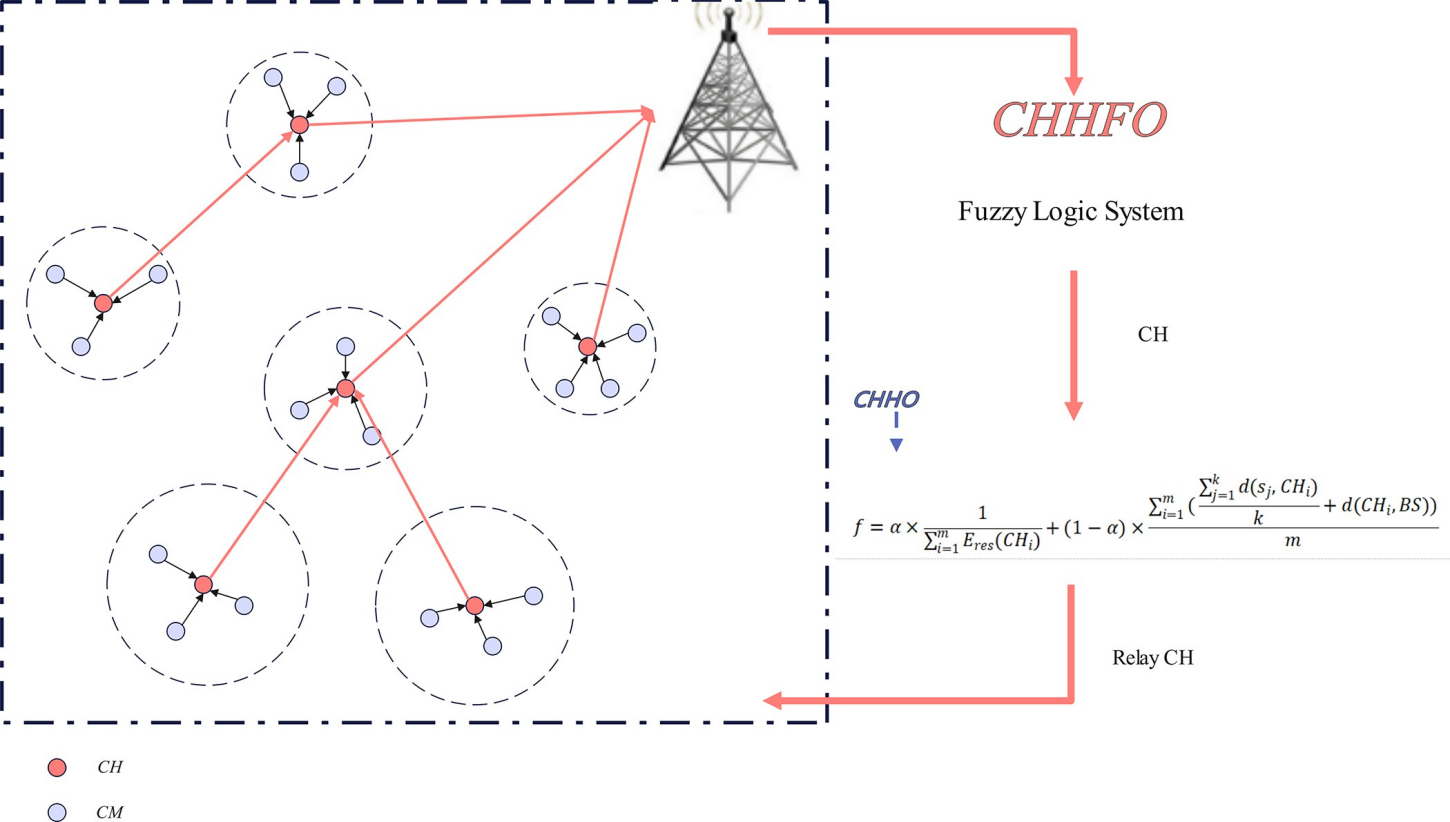
The framework of the proposed CHHFO protocol.

The effectiveness and endurance of wireless sensor networks rely on their adeptness in managing energy consumption. Improved efficiency in sensor energy utilization can significantly extend both the network’s lifetime and the nodes’ energy efficiency. Clustering stands widely acknowledged as a strategy to enhance network performance. CHHFO can leverage fuzzy logic and collaborative Harris Hawk optimization to bolster the network’s longevity and performance. Within the CHHFO protocol, fuzzy logic aids in cluster head selection, utilizing inputs like initial energy, neighboring nodes count, and distance from the base station to determine the most efficient CH. The fuzzy logic model assigns probability values to nodes involved in CH selection, where the highest-probability node contends for CH status. Data transmission from CH to BS occurs through multi-hop communication, facilitated by this protocol. The collaborative Harris Hawk Optimization algorithm plays a role in relay node selection and determines nodes’ fitness values, thereby conserving energy during data transmission. The relay node, possessing the highest fitness value, is tasked with transferring data to the BS. The CHHFO protocol framework is illustrated in Fig 2.

### 4.1. CH selection

**Fig 3:**
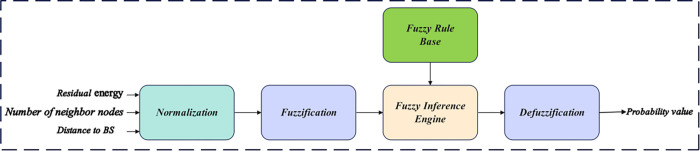
Fuzzy logic model used in the proposed protocol.

The nodes are clustered using a fuzzy logic model, assigning a CH to each cluster. Similar to [29], this scheme’s fuzzy model employs input variables to determine the most suitable node for the CH role. Utilizing node residual energy, number of neighboring nodes, and distance to the BS as inputs, the fuzzy system generates a probability value for each node in CH selection. CH selection hinges upon this probability value, electing the node with the highest probability as the CH. Thus, nodes with higher residual energy, closer proximity to the BS, and more neighboring nodes are deemed better suited for the CH role. The implementation of the fuzzy model simplifies the CH selection process, ensuring the selection of the most efficient node as the CH, thereby reducing uncertainty and enhancing the network’s overall lifetime and performance metrics. The components constituting the fuzzy model in the proposed protocol are detailed in Fig 3.

#### 4.1.1 Input

In a fuzzy logic system, the inputs typically consist of fuzzified variables or information received by the system. These inputs are commonly fuzzy quantized data, depicted as fuzzy sets, where each input variable generally encompasses one or multiple fuzzy sets. An affiliation function is employed to delineate the degree of association between the input values and these sets.

a. Residual energy

The protocol prioritizes residual energy as the pivotal factor in the Cluster Head selection process. Choosing nodes with substantial residual energy as cluster heads serves to prolong the network’s lifetime, sustain an extended active state, and foster a more stable connectivity, thus mitigating the risk of node failures or offline scenarios due to energy depletion. Furthermore, a stable cluster head aids in preserving network topology stability, averting instability arising from frequent turnover of cluster heads. Additionally, opting for nodes with higher residual energy as cluster heads forestalls premature energy exhaustion in individual nodes, thereby preventing localized performance degradation or coverage loss. The computation of a node’s residual energy is detailed in Eq 4 [11].


$${Input}_{1}=\sum_{i-1}^{N}\frac{E_{0}}{E_{0}-S(i).E}$$ (4)

**Fig 4:**
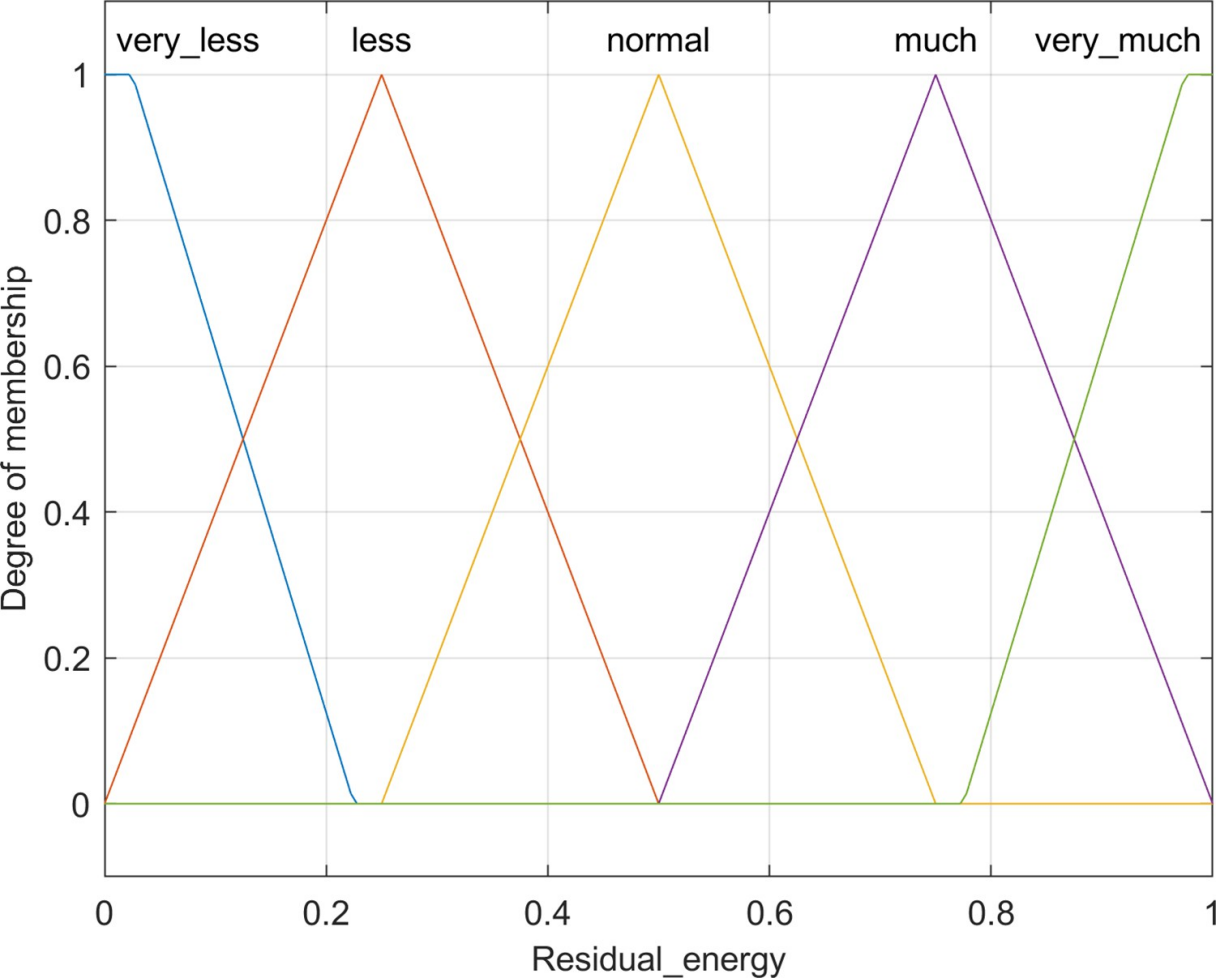
Membership function for residual energy.

Where *N* denotes the total nodes, *S(i)*.*E* is the current energy of node *i*, and *E*_0_ symbolizes the initial energy of nodes. Due to its importance for CH finding, three triangular and two trapezoidal membership functions are considered for residual energy, namely *very less*, *less*, *normal*, *much*, *very much*, which is shown in Fig 4.

b. Number of neighboring nodes

**Fig 5:**
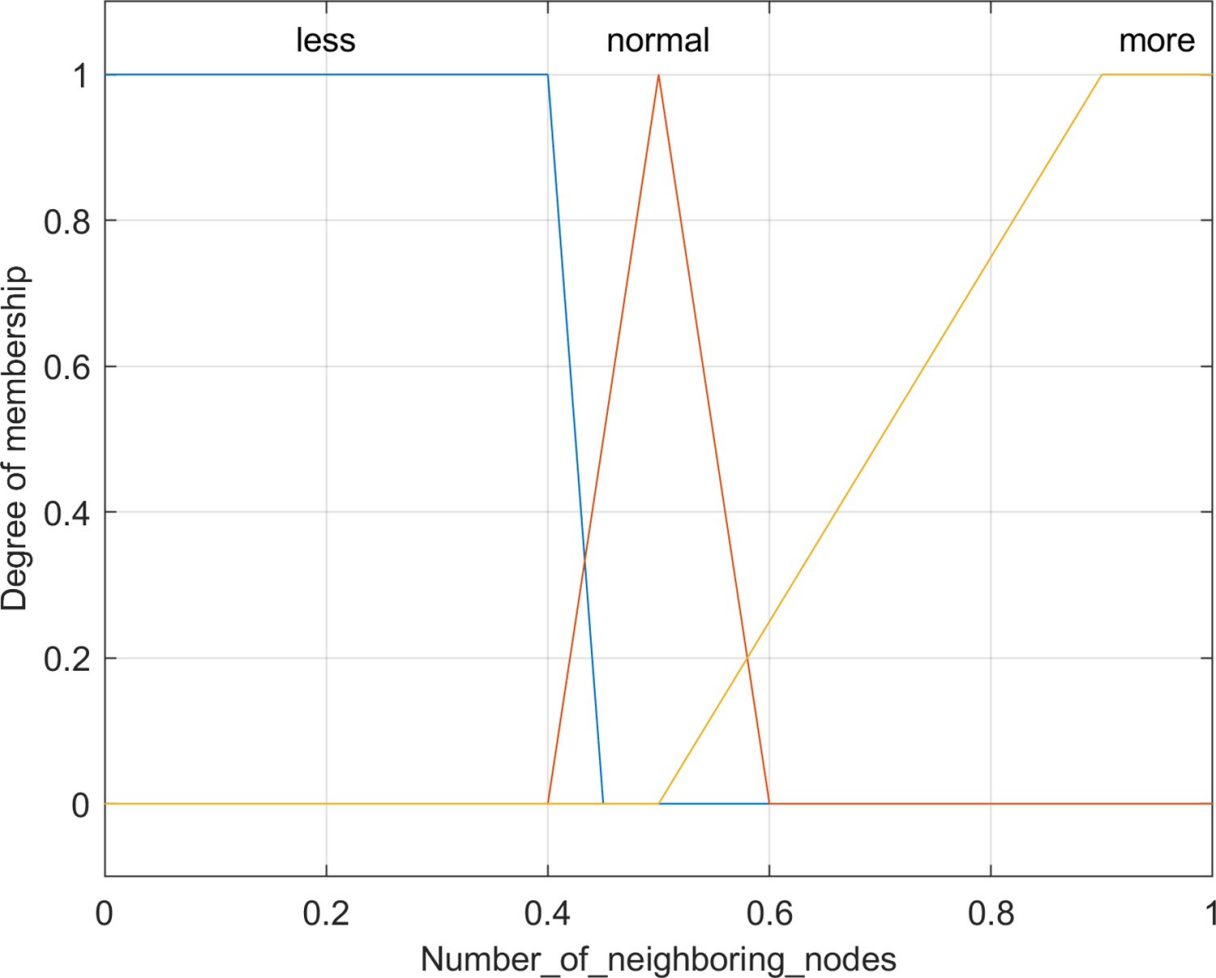
Membership function for the number of neighbor nodes.

The selection of Cluster Heads heavily relies on the count of neighboring nodes, a pivotal factor in enhancing data transmission efficiency. Nodes with a higher number of neighbors, when appointed as cluster heads, demonstrate superior capabilities in aggregating, processing, and transmitting data, significantly improving data transmission speed and efficacy. Additionally, the quantity of neighbor nodes reflects the topological layout and communication dynamics around each node, influencing network connectivity and communication efficiency. Opting for nodes with an increased count of neighbors as cluster heads not only strengthens connectivity but also fosters better collaboration, data forwarding, and network stability, thereby contributing to a more robust network structure. Concurrently, the role of a cluster head involves crucial tasks such as data aggregation, processing, and forwarding. Assigning a node with a greater number of neighboring nodes as the cluster head distributes the data transmission load, alleviating pressure on individual nodes within the network. Leveraging the assistance of neighboring nodes, the cluster head efficiently manages data transmission, reducing its workload, and ultimately elevating the overall network’s efficiency. Fig 5 portrays triangular and two trapezoidal membership functions, representing varying ranges of neighboring nodes: *less*, *normal*, and *more*. The computation of a node’s number of neighboring nodes is detailed in Eq 5 [15].


$${Input}_{2}=\sum_{i-1}^{N}neighbor\;count$$ (5)

c. Distance to BS

Proximity to the base station is considered an input factor. Nodes in closer proximity to the base station enable faster data transmission, thereby diminishing transmission delays and enhancing efficiency. Additionally, nodes nearer to the base station operate as cluster heads, transmitting data with reduced power and minimized signal loss. This proximity ensures higher reliability and stability in data transmission owing to decreased signal attenuation. Furthermore, choosing nodes near the base station as cluster heads aids in balancing energy consumption. Given their reduced energy consumption during data transmission, these nodes can evenly distribute the energy load, preventing localized energy depletion and effectively averting hot spot issues. The computation of a node’s distance to BS is detailed in Eq 6 [11,15].


$${Input}_{3}=\sum_{i=1}^{N}\sqrt{{\left(X_{BS}-X_{i}\right)}^{2}+{\left(Y_{BS}-Y_{i}\right)}^{2}}$$ (6)

**Fig 6:**
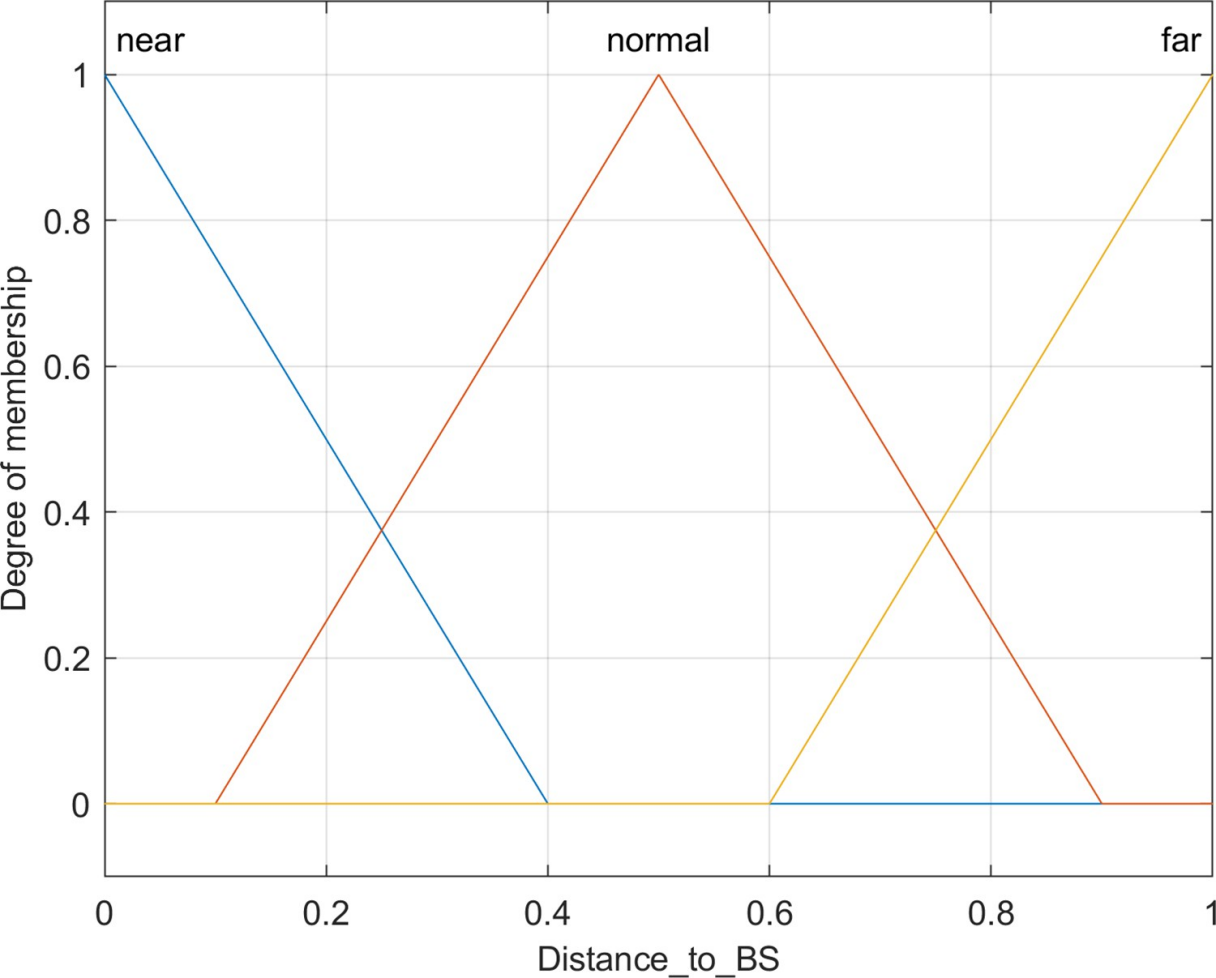
Membership function for the distance to BS.

The coordinates of the base station are (*X*_*BS*_, *Y*_*BS*_), the coordinates of node *i* are (*X*_*i*_, *Y*_*i*_), and the affiliation function is shown in Fig 6, represented by three triangular affiliation curves for *near*, *normal* and *far*.

#### 4.1.2 Normalization

In constructing a fuzzy logic system, normalization is typically executed to render input variables or data into a uniform range, ensuring data consistency. Subsequently, fuzzification is applied to map crisp values to a fuzzy affiliation function. These two steps collectively form the foundation for a fuzzy logic system, enabling effective handling of uncertainty and fuzzy data. Normalization effectively diminishes computational complexity and mitigates issues arising from disparate data ranges, thereby enhancing model stability and ensuring both the stability and accuracy of the fuzzification and fuzzy inference processes. This paper utilizes Min-Max Scaling to linearly map energy, distance, and the number of nodes to a range between 0 and 1. The normalization calculation process is as follows [15]:

$$X_{input}=\frac{X_{i}-X_{min}}{X_{max}-X_{min}}$$ (7)

Where X_max_, X_min_ are the maximum and minimum values of the data respectively, Eq 7 linearly transforms the original data so that the minimum value maps to 0 and the maximum value maps to 1.

#### 4.1.3 Fuzzification and defuzzification

Fuzzification and defuzzification represent pivotal stages in the operation of fuzzy logic systems aimed at addressing ambiguity and uncertainty. The defuzzification phase serves to convert precise inputs into a degree of membership function within a fuzzy set, thereby elucidating the correlation between input values and fuzzy concepts. This conversion facilitates the system’s ability to efficiently manage imprecise and fuzzy data, offering a means to characterize fuzzy concepts and languages in real-world applications. Defuzzification denotes the process of translating fuzzy outputs, derived from fuzzy reasoning, into explicit and unambiguous output values. This transformation renders the outcomes of fuzzy reasoning into tangible, understandable, and actual values by consolidating information from fuzzy outputs or employing specific mathematical algorithms, such as computing the weighted average or determining the center of gravity within the fuzzy set. This procedure aims to render the results of a fuzzy logic system more operational and interpretable, thereby supporting decision-making processes or the application of control systems. These two steps operate synergistically, allowing the fuzzy logic system to effectively manage fuzzy and uncertain information, ultimately providing clear outputs for subsequent analysis or action.

#### 4.1.4 Fuzzy inference

Fuzzy inference, as a fundamental process within fuzzy logic systems, relies on fuzzified inputs and fuzzy rules to generate outputs through fuzzy logic operations and inference mechanisms. This intricate process encompasses the conversion of precise inputs into fuzzy sets through defuzzification, employing "*If-then*" rules within the fuzzy rule base. Fuzzy inference is contingent upon the affiliation value of fuzzy inputs and the logical relationships embedded within the rules. Subsequently, it synthesizes the final fuzzy output. Fuzzy inference stands as an effective method for decision support and control systems, facilitating the generation of coherent and interpretable fuzzy outputs despite facing imprecise inputs and intricate conditions.

#### 4.1.5 Output

**Fig 7:**
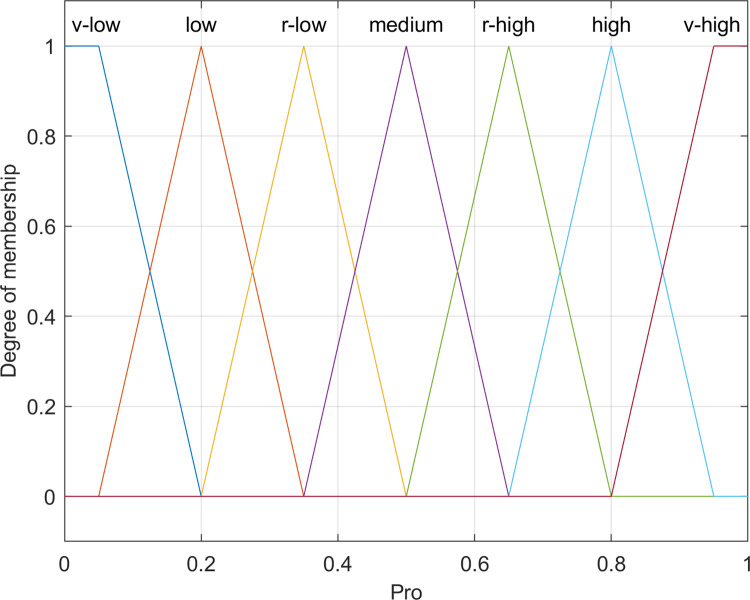
Membership function for probability value.

In the CHHFO protocol proposed herein, a fuzzy logic model is employed to determine the Cluster Head. This fuzzy inference system utilizes parameters including energy levels, neighbor count, and distance to the Base Station to generate an output in the form of a probability value. This value is derived through an affiliation function categorizing possibilities into *very low*, *low*, *rather low*, *normal*, *rather high*, *high*, and *very high*, which is depicted in Fig 7.

#### 4.1.6 Rule database

Rule Database is an important part of fuzzy logic system to store and organize fuzzy rules. It contains a series of "*If-then*" rules that describe the association between input variables and output variables. The rules used in CHHFO are 5*3*3 = 45 rules, and the rules established based on expert knowledge are shown in Table 2.

**Table 2:** Fuzzy rules.

*Input variables*				*Output*
	*Residual energy*	*Number of neighboring nodes*	*Distance to BS*	*probability value*
*1*	*Very less*	*less*	*far*	*Very low*
*2*	*Very less*	*less*	*normal*	*Very low*
*3*	*Very less*	*less*	*near*	*low*
*4*	*Very less*	*normal*	*far*	*low*
*5*	*Very less*	*normal*	*normal*	*rather low*
**.* *.* *.**	**.* *.* *.**	**.* *.* *.**	**.* *.* *.**	**.* *.* *.**
*45*	*Very much*	*more*	*near*	*Very high*

As depicted in Fig 3, the fuzzy inference system ingests parameters such as residual energy, neighbor count, and distance to the base station, subsequently generating a probability value as an output. In the process of CH selection guided by this probabilistic metric, the node with the highest probability is designated as the CH. Following the derivation of the probability value by the fuzzy system, each node transmits a message containing its unique *ID* and the associated probability to all nodes within its communication range. Upon receiving these messages with node *IDs* and associated probabilities, the nodes execute a selection process, identifying the node with the highest probability as the CH.

Following this, the CH broadcasts an announcement message to notify all nodes about the outcome of CH selection and the establishment of clusters. Non-CH nodes converge around the CH to configure clusters. The CH institutes Time Division Multiple Access (TDMA) scheduling to facilitate communication among cluster members and CHs, thereby mitigating communication conflicts. This procedural approach fosters efficient collaboration among network nodes concerning energy utilization and communication efficacy, ensuring seamless intra-cluster communication.

### 4.2. Relay finding

**Fig 8:**
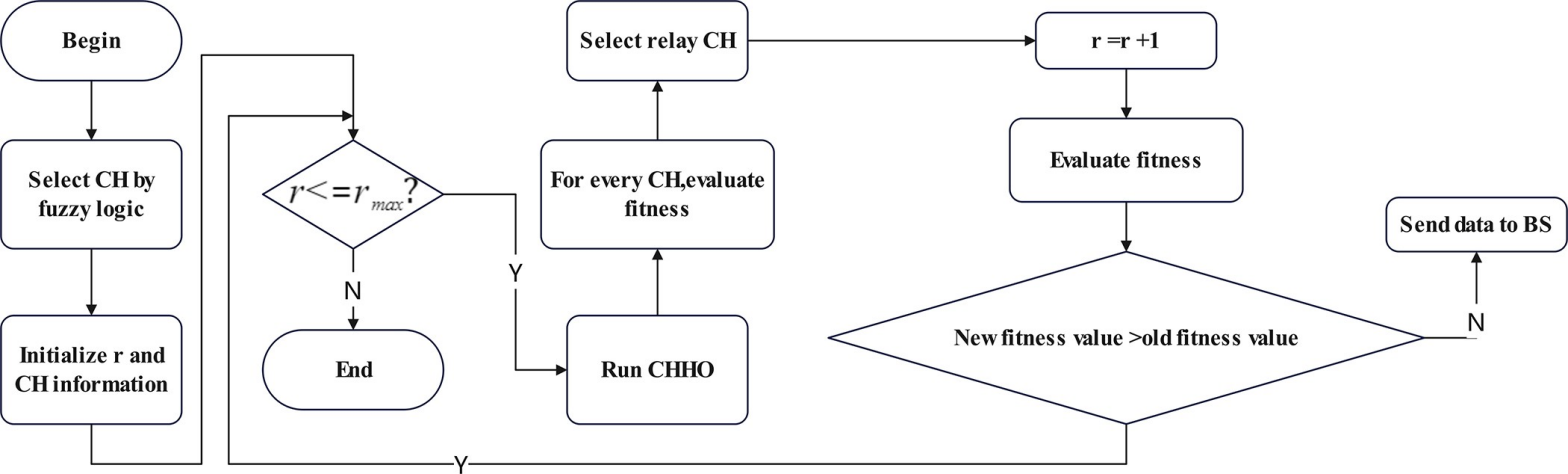
Flowchart of CHHO data transmission.

In the CHHFO protocol, subsequent to the selection of CH nodes via the Mamdani fuzzy system, data is relayed from these CHs to the BS in a multi-hop transmission mode. Certain CH nodes function as relay nodes, optimizing energy conservation by avoiding direct communication with the base station. The CHHO algorithm specifically identifies relay nodes among the selected CHs. Utilizing the CHHO technique, a fitness function is formulated to designate CH nodes for relay tasks, factoring in both the residual energy of the CHs and their proximity to the BS. Each CH node considered for relay selection undergoes fitness calculation through CHHO. During relay selection, the CH node exhibiting the highest fitness value assumes the role of a relay, facilitating the transmission of data from the CH node to the BS through a multi-hop pathway. The flowchart of the CHHFO is shown in Fig 8.

Every CH node undergoes assessment through a fitness function that accounts for both energy levels and distance metrics. Emphasizing high residual energy stands as the chief criterion in the selection of relay CHs, crucial for managing the added energy consumption during data aggregation and forwarding. Furthermore, the average distance within and among clusters assumes significance as another pivotal factor; shorter distances correlate with reduced network energy consumption and heightened energy efficiency. Consequently, Eq 8 defines the fitness function evaluated by the CHHO algorithm. By integrating the residual energy of the node and its distance from CH and BS, as follows.

$$f=\alpha \times \frac{1}{\sum_{i=1}^{m}E_{res}({CH}_{i})}+(1-\alpha )\times \frac{\sum_{i=1}^{m}\left(\frac{\sum_{j=1}^{k}d(s_{j},{CH}_{i})}{k}+d({CH}_{i},BS)\right)}{m}$$ (8)

where *E*_*res*_(*CH*_*i*_) denotes the residual energy of CH *i*, *d*(*s*_*j*_, *CH*_*i*_) and *d*(*CH*_*i*_, *BS*) indicate the distances from CM *j* to CH *i*, and from CH *i* to the BS, respectively. *k* is the number of CMs in the corresponding cluster, and α is the co-efficiency ranging in [0, 1] used to regulate the factors of energy and distance. In order to obtain the residual energy of the nodes, energy model as the same in [9,35] is utilized to calculate the energy consumption of data transmission with *k* bits over distance *d* between any two nodes *i* and *j*, and the energy consumption for data transmitting and receiving is expressed in Eqs (1) and (2), respectively.

In CHHFO, a Harris Hawk Optimization algorithm incorporating collaborative behaviour is proposed to select the best relay CH without too much information exchange between nodes.The Harris Hawk optimization algorithm is a new algorithm inspired from the hunting behavior of Harris Hawk. Exploration and exploitation are the two phases of HHO, which consider the perching behavior and pouncing mode of Harris hawk, respectively. A candidate relay CH is termed as a hawk, and its position is denoted by *x*_*ij*_ (*i* is the size of population, and *j* is the dimension of the solution), then the set of relay CHs is the solution as a prey expressed by $H_{i}=\{x_{i1},x_{i2},\ldots ,x_{ik}\}$, where *k* is the number of relay CHs. According to Eq 8, the higher the fitness value, the better the location of the Hawk. In other words, the better the selected relay node. Accordingly, the best individual can be determined as *H*_*prey*_ represented by $H_{prey}=\{x_{prey1},x_{prey2},\ldots ,x_{preyk}\}$ with the highest fitness value. Then the exploration or exploitation phase is applied to find the best solution determined by the escaping energy of prey expressed in Eq (9) [21,22].

$$E=2E_{\mathrm{init}}(1-\frac{t}{T})$$ (9)

where *E*_*init*_ is the initial energy of the prey randomly arranged in (-1, 1), *t* and *T* denote the current and maximum number of iterations, respectively. Once |*E*|≥1, the exploration phase continues to chase the prey and make it exhaust and lose all its energy, and the positions of the harks are updated by Eqs (10) and (11):

$$x_{\mathrm{ij}}^{t+1}=\{\begin{array}{l} x_{\mathrm{rand}}-r_{1}*|x_{\mathrm{rand}}-2r_{2}x_{ij}^{t}|,q\geq 0.5\\ (x_{\mathrm{preyj}}^{t}-x_{im}^{t})-r_{3}(lb+r_{4}(ub-lb)),q<0.5 \end{array}$$ (10)

$$x_{im}^{t}=\frac{\sum_{j=1}^{k}x_{i,j}}{k}$$ (11)

where *r*_1_, *r*_2_, *r*_3_, *r*_4_, *q* are random numbers between (0, 1), $x_{preyj}^{t}$ represents the position of the prey in current iteration, and *x*_*rand*_ denotes a randomly selected position. When |*E*|<1 after several iterations performed for exploration, the exploitation phase is started to choose and kill the prey by hawks. To avoid premature convergence, different exploitation strategies are used for various hunting situations based on *E* and *γ*(a random number between (0, 1), which consist of soft surround, hard surround, soft surround with progressive rapid dives, and hard surround with progressive rapid dives.

**Fig 9:**
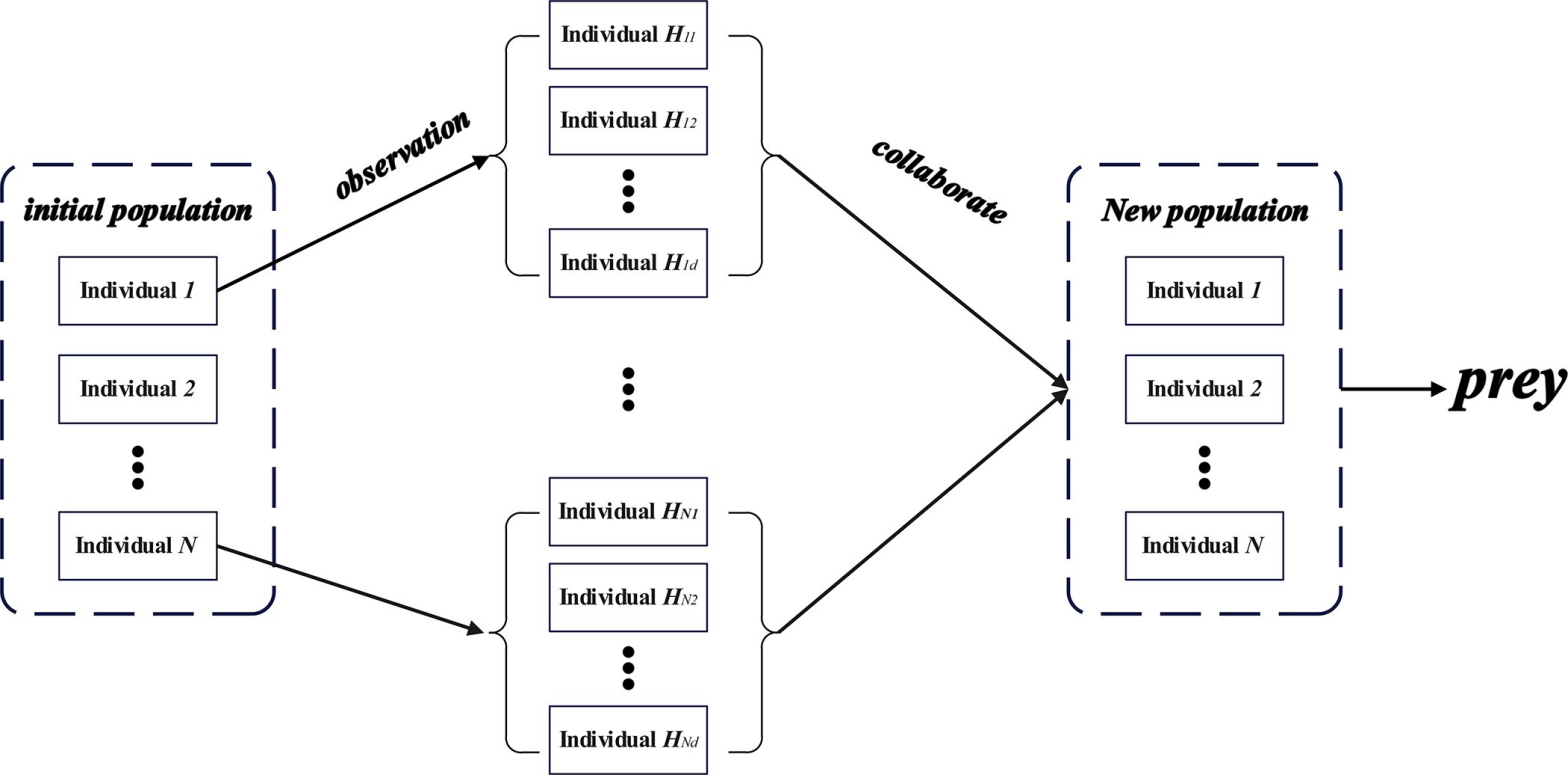
Localised exploratory phase collaborative process in CHHO.

The collaborative Harris Hawk algorithm uses the current global optimum *gbest* as a background variable, and in order to evaluate the goodness of the *d*-th dimension of an individual, the *d*-th dimension of the background variable is replaced by the *d*-th dimension of the individual to obtain a new individual. In this synergistic approach the individual contributes to each dimension of the *gbest* and is able to obtain a better individual. Take the example of a four-dimensional function *f* = |*x*−*z*|^2^, where z = (10, 20, 30, 40). The global optimum is X* = *z*. Assuming that there are individuals *X*_*1*_ = (5, 19, 24, 30) and *X*_*2*_ = (10, 16, 24, 27) in the current population, the *t—1* iterations produce the current optimum *gbest* = (7, 14, 21, 28), which is: *f (X*_*1*_*)* = 484, *f (X*_*2*_*)* = 529, *f (gbest)* = 900. Since *f (X1)*<*f (gbest)*, and the position of *gbest* will be directly replaced by *X*_*1*_, it results that *X*_*2*_ will lose the valuable information contained in the following iteration, i.e., the current first dimension of *X*_*2*_ is valued at 10, while the first dimension of *gbest* gets a worse value of 5. If we add a synergistic approach, and use *gbest* as a background variable, and use *X*_*1*_ and *X*_*2*_ to evaluate each dimension of *gbest*, we can get: *f (X*_*1*_*)* = 484, *f (X*_*2*_*)* = 529, *f (gbest)* = 900. If we add the synergistic method, *gbest* is used as a background variable, and *X*_*1*_ and *X*_*2*_ are used to optimize each dimension of *gbest*, and the new better value generated becomes the new background variable, and then the next dimension is compared, until the last dimension is finished, and finally the best value after the synergistic method is obtained. In the first dimension, *X*_*1*_ generates *g*_*1*_ = (5,14,21,28) after synergising with *gbest*, *X*_*2*_ generates *g*_*2*_ = (10,14,21,28) after synergising with *gbest*, at this time, if *f (g2)*<*f (gbest)*<*f (g1)*, then *gbest* and *g*_*1*_ are discarded and *g*_*2*_ is chosen as the new background variable, i.e., *gbest* = g_2_, then the above process is repeated in the second dimension, until the end of the last dimension, the optimal value is obtained. Repeat the above process in the second dimension, and finally get the more accurate global optimum *gbest* = (10, 19, 24, 30). More accurate results are obtained after such a collaborative process of calculation. The Localised exploratory phase collaborative process are shown in Fig 9.

***1) Soft surround (E ≥ 0.5*, *γ* ≥ 0.5)**

In this situation, the prey has sufficient energy to escape the hunt, because the harks locate the prey from a long distance, so the hawks try to encircle the prey to make it exhausted for a surprise pounce upon it. Therefore, the positions are updated as follows.


$$x_{ij}^{t+1}=\Delta x_{ij}^{t}-E^{*}|J^{*}x_{\mathrm{preyj}}^{t}-x_{ij}^{t}|$$ (12)

$$\Delta x_{ij}^{t}=x_{\mathrm{preyj}}^{t}-x_{ij}^{t}$$ (13)

Where *J* = 2*(1−*r*_5_) indicates the strength for escaping from the hawks of the prey, and *r*_5_ is a random number between (0, 1).

***2) Hard surround (E ≥ 0.5*, *γ* ≥ 0.5)**

In this case, the prey has no energy to escape from the catch performed by the harks or well positioned for the hawks to make a quick and successful attack. Here, the positions are updated in this strategy according to Eq (14).


$$x_{ij}^{t+1}=x_{\mathrm{prevj}}^{t}-E^{*}|\Delta x_{ij}^{t}|$$ (14)

***3) Soft surround with progressive rapid dives (E ≥ 0.5*, *γ* < 0.5)**

In this strategy, the prey has enough energy to void being caught by the hawks, and the hawks enforces soft surround before making progressive dives. Moreover, Lévy flight is used to implement progressive movement towards the already identified promising local region. Hence, the positions are updated by using Eq (15).

$$x_{ij}^{t+1}=\{\begin{array}{l} Y:x_{\mathrm{preyj}}^{t}-E^{*}|J^{*}x_{\mathrm{preyj}}^{t}-x_{ij}^{t}|,\mathrm{if}\;F(Y)<F(H_{i}^{t})\\ Z:Y+S^{*}LF(D),\mathrm{if}\;F(Z)<F(H_{i}^{t}) \end{array}$$ (15)

where *S* is a *D*-dimensional random row vector, $LF(D)=0.01*\frac{u^{*}\delta }{|v{|}^{\frac{1}{\beta }}}$ is Lévy flight function in which standard deviation *δ* can be expressed as follows.


$$\delta ={\left(\frac{\Gamma (1+\beta )*\sin(\frac{\pi \beta }{2})}{\Gamma (\frac{1+\beta }{2})*\beta *2(\frac{\beta -1}{2})}\right)}^{\frac{1}{\beta }}$$ (16)

The two random variables *u* and *v* in the Lévy flight function ranges between (0, 1), and *β* is a constant whose value is 1.5.

***4) Hard surround with progressive rapid dives (E < 0.5*, *γ* < 0.5)**

In this instance, the prey can escape from the hunt from the hawks though it has no energy. The hawks perceive the situation not suitable enough for performing the catch, so it tries to get closer to the prey so as to apply a hard surround before making progressive dives.


$$x_{ij}^{t+1}=\{\begin{array}{l} Y:x_{\mathrm{preyj}}^{t}-E^{*}|J^{*}x_{\mathrm{preyj}}^{t}-x_{im}^{t}|,\mathrm{if}\;F(Y)<F(H_{i}^{t})\\ Z:Y+S^{*}LF(D),\mathrm{if}\;F(Z)<F(H_{i}^{t}) \end{array}$$ (17)

In addition, the neighborhood centroid opposition based learning mechanism is utilized to expand the search space and avoid falling into local optima. For an individual $H_{i}=\{x_{i1},x_{i2},\ldots ,x_{ik}\}$, its neighborhood centroid opposition is given by:

$$H_{i}^{*}=\{{(2*Mi-x}_{i1}),(2*Mi-x_{i2}),\ldots ,(2*Mi-x_{ik})\}$$ (18)

$M_{i}=\frac{\sum_{j=1}^{k}x_{ij}}{k}$ indicates the centroid of a local region. Moreover, the search space is constrained in range *(min(x*_*ij*_*)*, *max(x*_*ij*_*))*. Once beyond the boundary, calculate the opposition using Eq (19).


$$x_{ij}^{*}=\left\{ \begin{array}{l} x_{ij}+rand()\times (M_{i}-min(x_{ij})),if\;x_{ij}^{*}<x_{ij}\\ M_{i}+rand()\times (max(x_{ij})-M_{i}),if\;x_{ij}^{*}>x_{ij} \end{array}\right.$$ (19)

Furthermore, the individual is updated to $H_{i}^{*}$ in the next iteration when $F(H_{i}^{*})<F(H_{i})$. When *t* reaches the maximum number of iterations, the best solution is achieved, which is the individual of hawks with the optimal fitness value. This individual maps the best relay selected by CHHO. All the data collected by the CH is sent to the nearest relay node, which then forwards the received data to the next relay node on the path to the BS and so on until it reaches the BS.

## 5. Simulation results

The experiments were conducted on a computer running the Windows 10 operating system, equipped with an AMD Ryzen 5 3500X 6-Core processor, 16GB of RAM, and a 500GB SSD. The MATLAB R2022a platform is used to simulate and test the performance of the proposed CHHFO. To ensure the robustness of the results, we repeated the experiments 50 times and calculated the average to draw the conclusions.

In order to strictly verify the performance of the proposed CHHFO protocol, we conducted a comparative analysis. The analysis involves the comparison of the protocol EFCR [31] using fuzzy systems, the intelligent algorithm HHO-UCRA [33] and the protocol IHHO-F [36] combining intelligent algorithms with fuzzy systems. These comparisons are carried out in the same simulation environment. In addition, the selected intelligent algorithms are HHO and its improvements.

### 5.1. Simulation settings

To comprehensively assess the effectiveness of the CHHFO protocol, we adopted standard simulation parameters consistent with prevalent protocols, alongside network and radio energy models outlined in Table 3. In this investigation, CHHFO undergoes comparison against EFCR, HHO-UCRA, and IHHO-F to assess its throughput, scalability, and network lifetime across three scenarios. These scenarios are set up in three main directions, where the variation of the number of nodes 100, 200 and 400 focuses on the testing of the throughput of the proposed CHHFO protocol, whereas the expansion of the network area size from 200m * 200m, 400m * 400m to 800m * 800m intuitively reflects the network scalability through the number of surviving nodes, and the variation of the location of the network area in which the base station is situated focuses on the observation of the energy consumption of the network and network lifetime.

**Table 3:** Simulation parameters.

*Parameters*	*Scenario 1*			*Scenario 2*			*Scenario 3*		
*Number of nodes*	***100***	***200***	***400***	*100*			*100*		
*Initial energy*	*0*.*5J*			*0*.*5J*			*0*.*5J*		
*E*_*elec*_	*50 (nJ/bit)*			*50 (nJ/bit)*			*50 (nJ/bit)*		
*E*_0_	*5(nJ/bit)*			*5(nJ/bit)*			*5(nJ/bit)*		
*ε*_*fs*_	*10 (pJ/bit/m* ^*2*^ *)*			*10 (pJ/bit/m* ^*2*^ *)*			*10 (pJ/bit/m* ^*2*^ *)*		
*ε*_*mp*_	*0*.*0013 (pJ/bit/m*^*4*^*)*			*0*.*0013 (pJ/bit/m*^*4*^*)*			*0*.*0013 (pJ/bit/m*^*4*^*)*		
*d*_0_	*87*.*7m*			*87*.*7m*			*87*.*7m*		
*Data packet size*	*4000bits*			*4000bits*			*4000bits*		
*Control packet size*	*200bits*			*200bits*			*200bits*		
*BS Location*	*x = 100m*,*y = 100m*			***x = 100m*,*y = 100m***	***x = 200m*,*y = 200m***	***x = 400m*,*y = 400m***	***x = 100m*,*y = 100m***	***x = 100m*,*y = 200m***	***x = 100m*,*y = 250m***
*Network area*	200m*200m			200m*200m	400m*400m	800m*800m	200m*200m		

### 5.2. imulation result and discussion

#### 5.2.1 Scenario 1

**Fig 10:**
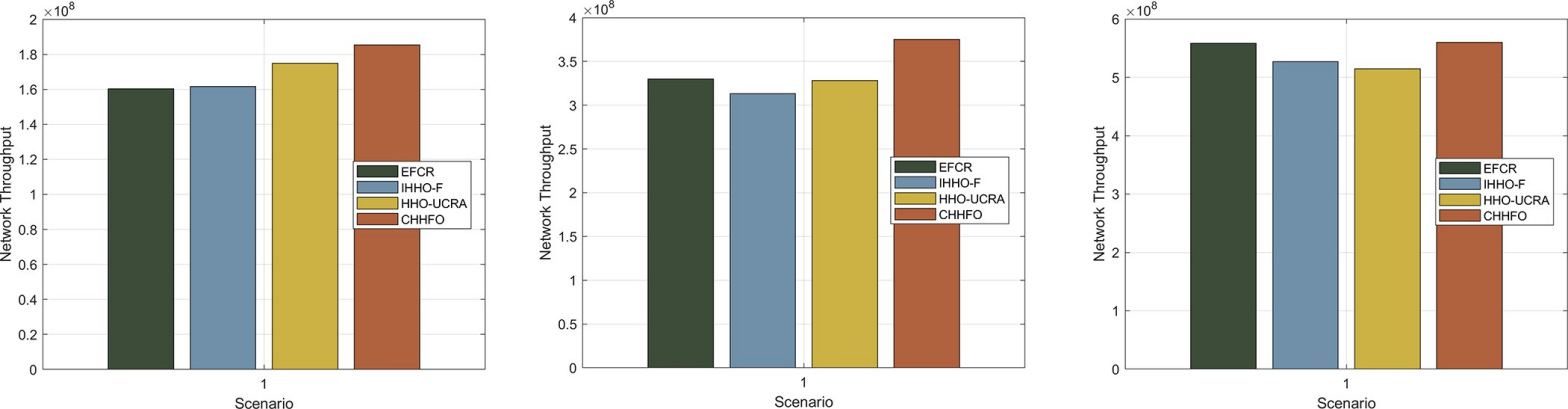
Comparison of the network throughput. (a) Number of nodes 100; (b) Number of nodes 200; (c) Number of nodes 400.

Throughput represents the volume of data or information effectively transmitted within a specific time frame, serving as a critical performance metric for networks. Typically, throughput serves as a standard metric to assess a network’s data transmission capability and overall performance. Improved throughput substantially enhances network performance, diminishes transmission latency, and broadens the spectrum of supported application scenarios encompassing monitoring, control, and data transfer. In Scenario 1, we assessed the throughput of the proposed CHHFO protocol by varying the number of nodes, maintaining consistent network area size, and base station placement. Fig 10 displays the network throughput test results.

Fig 10 illustrates an intuitive relationship: throughput grows as the number of nodes increases. Across the three scenarios, CHHFO consistently demonstrates notably superior network throughput compared to EFCR, IHHO-F, and HHO-UCRA. This stems from CHHFO’s utilization of fuzzy logic and the collaborative Harris Hawk algorithm, employed in clustering and routing phases for data transmission. CHHFO enhances network throughput by 13.53%, 12.07%, and 0.32% in comparison to EFCR, and by 12.77%, 16.47%, and 5.96% in contrast to IHHO-F. Moreover, it surpasses HHO-UCRA by 5.66%, 12.48%, and 8.16%, respectively. In the case of EFCR, while CH is selected based on demand among two clustering algorithms, there’s a deficiency in adapting the routing component. IHHO-F likely experiences low network throughput due to heightened energy consumption and a shortened network life cycle caused by a high SCH load. HHO-UCRA’s performance is relatively sound as it determines optimal clusters and routing paths through HHO. However, an energy consumption imbalance emerges with increasing node count.

#### 5.2.2 Scenario 2

**Fig 11:**
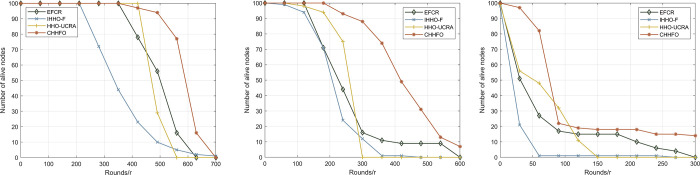
Comparison of the number of alive nodes. (a) Area size is 200m*200m; (b) Area size is 400m*400m; (c) Area size is 800m*800m.

Alongside throughput, the scalability of the protocol holds significant importance due to the widespread utilization of wireless sensor networks across diverse applications for monitoring and data collection purposes. Robust scalability in cluster and routing protocols enables enhanced adaptability to network expansion and management changes while ensuring sustained network performance, energy efficiency, and data transmission quality. Within scenario 2, we vary the network area size to assess protocol scalability, specifically by examining the incidence of dead nodes. Table 4 and Fig 11 depict the network lifetime outcomes of the CHHFO protocol across regions measuring 200m*200m, 400m*400m, and 800m*800m, each centered around the Base Station.

**Table 4:** Comparison of FND, HND and LND.

		*CHHFO*	*HHO-UCRA*	*IHHO-F*	*EFCR*
200m*200m	*FND*	***380***	*449*	*250*	*359*
	*HND*	***573***	*482*	*331*	*504*
	*LND*	***689***	*543*	*754*	*630*
400m*400m	*FND*	***191***	*90*	*42*	*144*
	*HND*	***418***	*255*	*201*	*225*
	*LND*	***628***	*285*	*431*	*579*
800m*800m	*FND*	***26***	*1*	*1*	*4*
	*HND*	***73***	*47*	*16*	*32*
	*LND*	***549***	*147*	*248*	*275*

Fig 11 illustrates that as the network area expands, IHHO-F experiences premature failure in the routing stage caused by node overload. In contrast, HHO-UCRA progressively demonstrates its advantages by employing the HHO algorithm and selecting energy-efficient routing schemes. The CHHFO protocol we propose integrates energy consumption considerations into both the clustering and routing phases, enabling it to select optimal solutions at each stage and exhibit superior scalability compared to alternative protocols.

#### 5.2.3 Scenario 3

**Fig 12:**
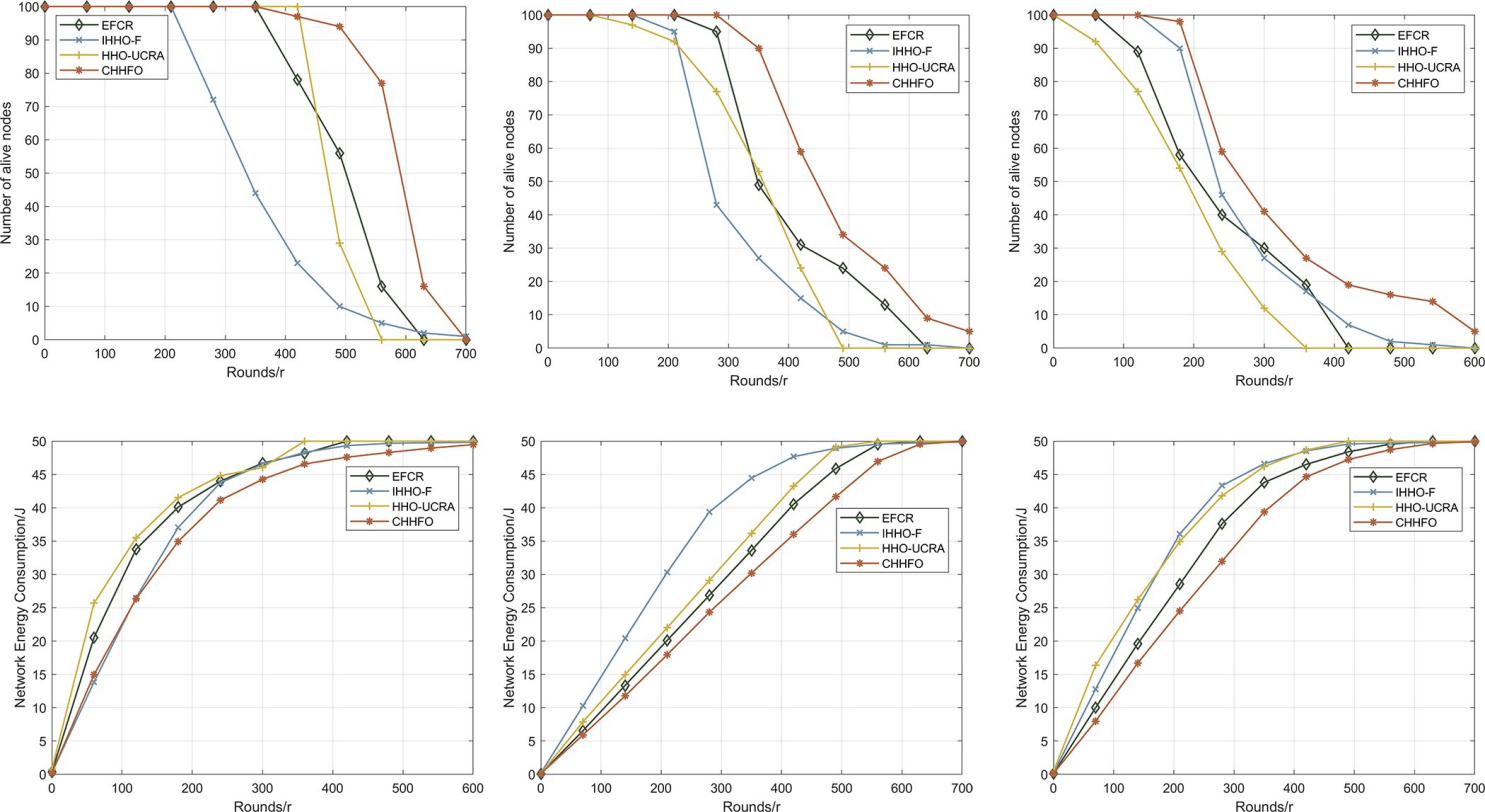
Comparison of the number of alive nodes. (a) BS coordinates is (100,100); (b) BS coordinates is (100,200); (c) BS coordinates is (100,250). Comparison of the network energy consumption. (d) BS coordinates is (100,100); (e) BS coordinates is (100,200); (f) BS coordinates is (100,250).

In wireless sensor networks, assessing the network lifetime is paramount, defining the duration during which the entire network can maintain efficient operation. It signifies the duration within which the network operates before its sensor nodes exhaust their energy reserves. Given that wireless sensor nodes rely on batteries with finite lifetimes, efficient energy management becomes crucial for prolonging the network’s lifetime. Scenario 3 assesses the network lifetime based on both the count of defunct nodes and the overall network energy consumption. Fig 12 illustrates the comparative outcomes between CHHFO, EFCR, IHHO-F, and HHO-UCRA.

Fig 12 demonstrates that CHHFO consistently outperforms other algorithms, despite variations in the placement of Base Stations. Regarding IHHO-F, while it considers energy, distance, and the count of neighboring nodes for optimal cluster head selection, excessive data transmission loads can prematurely deplete SCH, impacting the network’s overall lifetime. In HHO-UCRA, the selection of Cluster Heads by the Harris Hawk Optimization accounts for factors like neighboring node distance, distance to BS, and energy ratios. Moreover, it incorporates remaining energy, distance between source and candidate trunk CH, and candidate trunk CH’s node degree to determine data communication routes, effectively extending the network’s lifetime. However, CHs closer to BS in HHO-UCRA are prone to premature depletion due to increased data forwarding. EFCR employs a fuzzy logic system during the clustering stage but lacks a corresponding routing strategy for data transmission in wireless sensor networks. In contrast, CHHFO initially employs a fuzzy logic system to select optimal CHs and then employs a collaborative Harris Hawk algorithm to locate relay nodes. Although the network’s energy consumption rises with the number of turns, CHHFO’s energy distribution remains significantly lower compared to other protocols. Specifically, when energy consumption reaches half, CHHFO’s running rounds surpass IHHO-F by 39.79%, EFCR by 34.25%, and HHO-UCRA by 0.88%. These findings underscore CHHFO’s substantial energy efficiency improvements over other protocols. In essence, CHHFO considers residual energy, distance, energy standard differences, and employs the fuzzy logic system’s distribution characteristics to maintain a balance among these parameters, ultimately elongating the network’s lifetime.

## 6. Conclusion

This paper presents CHHFO, a novel clustering and routing protocol aimed at enhancing network energy efficiency and prolonging network lifetime. The protocol prioritizes energy efficiency and equilibrium during cluster formation and relay node determination, implemented through fuzzy logic and the collaborative Harris Hawk algorithm. According to the results, the network throughput, HHOCFR is 8.76%, 11.73%, 8.64% higher than HHO-UCRA, IHHO-F, and EFCR. In addition, he energy consumption of HHOCFR is lower than HHO-UCRA, IHHO-F, and EFCR by 0.88%, 39.79%, 34.25%, respectively. Moreover, CHHFO exhibits superior scalability compared to other protocols. However, methods such as intelligent algorithms and fuzzy logic systems alone cannot meet the needs of dynamic networks. Therefore, in future research, we will combine deep reinforcement learning technology with wireless sensor networks to meet social needs.

## Supporting information

S1 File (XLSX)

## Abbreviations

Terminology WSN: *Wireless Sensor Networks*
CH: *Cluster Heads*
BS: *Base Station*
HHO: *Harris Hawk Optimization*
CHHFO: *Collaborative Harris Hawk Fuzzy Optimization*
FLS: *Fuzzy Logic System*
COA: Center Of Area
E_Tij_: *The energy required if a node i sends k-bit data to a node j far away from the distance d*
E_elec_: *The electronics energy consumption used for transmitting or receiving 1-bit data*
Ε_fs_, ε_mp_: *The amplifier coefficients used for free space and multi-path model*
*d*_0_: The distance threshold for the model determination
E_Rij_: The energy consumed for k-bit data aggregation
E_DA_: The energy required for aggregating k-bit data
E_pDb_: The energy consumption for 1-bit data fusion
